# Research on subgroups is not research on equity attributes: Evidence from an overview of systematic reviews on vaccination

**DOI:** 10.1186/s12939-017-0587-x

**Published:** 2017-06-07

**Authors:** Xavier Bosch-Capblanch, Meike-Kathrin Zuske, Christian Auer

**Affiliations:** 1 Swiss Tropical and Public Health Institute, Socinstrasse 57, 4002 Basel, Switzerland; 2 Universität Basel, Petersplatz 1, 4003 Basel, Switzerland

## Abstract

**Background:**

Equity remains a priority in the international health development agenda. However, major inequities in vaccination coverage jeopardise the achievement of the Sustainable Development Goals. We aim at comprehensively describing how research has addressed equity issues related to vaccination.

**Methods:**

We carried out an overview of systematic reviews (SRs) that explicitly explored the effects of interventions to improve vaccination in any context; for any vaccine and, in any language. We followed standard research synthesis methods to systematically search for SR, assess them for inclusion and extracting relevant data, particularly on vaccination related outcomes. To gather evidence on equity issues addressed in the SR, we used the PROGRESS-plus framework.

**Findings:**

Our search obtained 2,003 hits which resulted in 54 included SRs, published between 1994 and 2014. The quality of SRs was generally poor, with less than half complying with most of the quality criteria. Reported vaccines included, by order of frequency, influenza and Expanded Programme on Immunisation vaccines. The types of interventions more frequently reported were related to vaccination delivery strategies, financial support and information, education and communication. Most of the SRs suggested effects favouring intervention groups as opposed to comparison groups. The most frequently reported equity attribute was ‘place of residence’ and the least reported equity attributes were sexual orientation and religion. Very few estimates of effects actually measured differences or changes between groups having those attributes and all of them referred to the place of residence. No data was found about reducing equity gaps for vulnerable groups or minorities, or attributes such as sexual orientation, education or specific religious groups.

**Conclusions:**

Although research on vulnerable populations as a subgroup is abundant, it fails to report on the interventions that will actually reduce inequities and consider how redistribution of health care resources could shrink the gap between the privileged and most vulnerable groups including minorities. Research, if aiming at being responsive to global health policy trends, needs to report not only on specific attributes but also on how a better redistribution of health care resources could contribute to alleviating the unjust situation of the most vulnerable populations.

**Electronic supplementary material:**

The online version of this article (doi:10.1186/s12939-017-0587-x) contains supplementary material, which is available to authorized users.

## Research in context

### Evidence before this study

We searched for published Systematic Reviews (SR) related to vaccination issues (e.g. Oyo-Ita 2011) and looked for evidence on interventions to reduce inequities. However, despite that the SR could focus on vulnerable populations, there was hardly any report on differential measures between groups to describe how equity could be improved. This lack of evidence on differentials would be a serious gap to informing global policies and their contribution to the Sustainable Development Goals.

### Added value of this study

In order to verify to which extent this lack of evidence was real, we carried out an overview of systematic reviews containing vaccination outcomes, scrutinising whether these reviews did or did not report on equity issues.

We confirmed that despite the wealth of research on vulnerable groups there were two gaps in research: (1) while some ‘generic’ groups of vulnerable populations (e.g. the poor) are widely studied, minorities and other well-known disadvantaged groups (e.g. low educational status) are not. (2) Research reports on the effects of interventions in some vulnerable groups but hardly on actually reducing differences between the privileged populations and vulnerable ones on how to better redistribute health care, hence reducing inequities.

### Implications of all available evidence

Standard research methods should be developed and encouragement given to use these to measure the effects of interventions that will potentially be useful in reducing inequity. Policy makers should demand better guidance not only on what works in vulnerable populations but, on how to be responsive to the unjust distribution of health and health care resources.

## Introduction

Equity in health is a fundamental moral and ethical commitment to reduce and eliminate unfair and unjust disparities in health and its determinants [1]. Health equity has been at the heart of international initiatives of human development for decades. [2]. With the recent declaration of the Sustainable Development Goals (SDG), communities, social entities, Non-Governmental Organisations, multi-lateral organisations, academia and other actors have repeatedly acknowledged the relevance of equity to ensure sustainable development [3]. Vaccination is no exception as it will target the whole population at several stages in life.

Data from WHO estimates based on administrative reporting and surveys, and data from large, good quality Demographic and Health Surveys, point at the same direction: while vaccination coverage of various antigens has steadily increased globally in the past decades, it has recently stagnated at 85% or 86% globally [4] (third dose of Diphtheria – Tetanus – Pertussis vaccine –DTP3; WHO estimates) and reached 76% [5] (DTP3 in 2015, WHO estimates) in the African Region. However, this progress has been unequal in different countries [6] and a considerable proportion of children remains partially or not fully vaccinated [7]. Factors like family income, education or geographic location are still associated with different vaccination coverage rates [8], especially in Low and Middle Income Countries (LMIC) [6]. For example, in Mali, immunisation is two times higher in wealthier households than compared to poor ones, with this gap widening over 30% in recent years (Demographic and Health Surveys) [9]. All these data invariable link low vaccination coverage with less wealth, being it at international, national or at sub-national levels.

One of the six guiding principles in the Global Vaccine Action Plan (GVAP) is equity: “equitable access to immunisation is a core component of the right to health”[10]; reducing disparities in immunisation, typically measured with vaccination rates, means that every eligible individual is vaccinated. Other principles in the GVAP refer to strategies consistent with the reduction of inequities; such as shared responsibility between vaccination partners and sustainability principles. There are four compelling arguments to reduce inequities in vaccination outcomes [11]. Firstly, access to vaccinations is a component that contributes to a person being assured of their human rights to health. Even for the more recently introduced vaccines [12] vaccines are a cost-effective intervention to reduce childhood morbidity and mortality. As such vaccinations (and health care in general) have to be seen as social and human assets which move in the direction of justice and equity. [13]. Thirdly, the potential benefit of reaching the unreached would be significantly higher as compared with the general population by making individuals and households healthier [10]; Lastly, other health care interventions can synergise with outreach and inreach vaccination programmes by mutually facilitating access to common population targets [14]. Strikingly, in a review of 62 vaccination country multi-year plans [15], we found few references to inequities in vaccination coverage, except for the case of Ethiopia which had clearly formulated an objective of reducing the percentage of unimmunised children.

Although there is already a variety of “universal” and “targeted” interventions to reduce inequities in vaccination, often reported in terms of coverage; evidence on the effectiveness of these interventions to reduce the gap between populations accessing services and those with substantial barriers to receiving health care seems inadequately addressed. Vaccination coverage has been used for decades to report vaccination programmes performance. Recent analyses suggest as well that coverage estimates (including vaccination coverage) are promising indicators to track health determinants nationally and internationally [16]. Several Cochrane reviews have examined the effects of interventions to improve vaccination coverage but without investigating or finding their effects across different population groups [17–19]. Målqvist looked at equity issues in targeted interventions in LMIC [20] but only in relation to measles.

While research describing the health status of vulnerable groups is abundant, research on interventions that impact on their health is less prominent. Yet, if health policies have to address inequities, evidence, not only on what works for the most vulnerable, but on what interventions can actually reduce the inequity gaps, is required.

Through an overview of systematic reviews (SRs) in the area of vaccination the aim of this work is to describe how globally research on the effects of interventions on vaccination coverage has targeted vulnerable populations, and particularly which research exists on interventions to reduce inequity gaps. We believe that the wealth of systematic reviews on the effects of interventions to improve vaccination outcomes should portray a comprehensive picture of past and current research on the topic.

## Methods

### 1) Criteria for considering systematic reviews for this overview

We conducted an overview of SRs. We searched for and included any SR aiming at describing the effects of interventions to improve vaccination related outcomes, without limitations in the type of participants (i.e. receivers of vaccination, caregivers, health care providers and managers), type of intervention, type of vaccine involved or outcome (i.e. morbi-mortality, coverage rates, behaviours, knowledge). We focused on vaccination coverage outcomes because these are widely used to report on vaccination programmes performance [21] in the understanding that inequities in vaccination coverage may reflect inequities on underlying issues (such as education, wealth or access to health care). Under the term ‘coverage’ we include as well, vaccination timeliness and completeness.

The tasks of deciding on relevance, applying the inclusion criteria and data extraction were singly done and distributed among co-authors of this article. Co-authors regularly cross-checked the decisions made in a subsample of SR, especially when potential inconsistencies were identified in the data extracted.

We decided that an article was an SR if it reported a search strategy, search terms, at least one literature source, inclusion criteria, types of participants and interventions. Reviews of a wider scope than that of vaccination (e.g. preventive measures in general) but containing vaccination data were equally considered.

We excluded systematic reviews which only included descriptive studies (e.g. results of Demographic and Health Surveys) because these studies do not provide findings on the effects of interventions to improve vaccination. However, if a systematic review contained studies which used Demographic and Health Surveys to report the effects of interventions, this was included.

### 2) Literature searches

We searched for published SRs, without limits on the year of publication and language, in the following electronic bibliographic databases: Medline, EMBASE, PsycInfo, Cochrane, Web of science, CINAHL and Ebsco; with terms related to vaccination, immunisation, systematic review and meta-analyses (and their variant terms). The original search was carried out in 2013 and was updated in November 2014 including ‘PDQ‐evidence’ [22], an online literature platform to search SRs and to link with primary studies. See Additional file 1 for an example of search strategy.

### 3) Data collection

Duplicate references were removed and studies were assessed for relevance using titles and abstracts. Full text relevant articles were screened using the inclusion criteria described. Data was extracted using a standard template and included reference information, SRs data (e.g. number of studies in the SR), participants, interventions, comparators, equity groups and effects estimates. We classified interventions into the following broad categories: delivery of care, financial, human resources, Information, Education and Communication (IEC), infrastructures, organisation and regulations and substance or device. Other interventions or combinations of interventions were categorised as ‘other’. Data was extracted as reported and we used data from individual studies when data was not pooled in SRs. Inclusion and data extraction were carried once with regular checks from the most senior authors and additional data checks were set up to verify extracted effect estimates. We considered data reported in any form: absolute differences, relative differences and changes in time of the intervention groups.

### 4) Consideration of equity in systematic reviews

Equity can be considered under two perspectives: as interventions specifically targeting vulnerable groups (‘targeted interventions’) or as interventions for the whole population but the effects of which are only measured in vulnerable groups [23]. However, it was hardly possible to identify this feature in the objectives of the SR or primary studies. Data on equity was extracted following PROGRESS‐plus [24], which stands for Place of residence; Race/ethnicity, Occupation, Gender, Religion, Education, Socioeconomic status (SES), Social capital, ‘plus’ age, disability and the sexual orientation. We also captured and categorised as ‘other’ attributes which reviews’ authors would relate to vulnerability (e.g. groups of subjects with chronic conditions). Where available we extracted differences or gradients of effects in equity groups.

### 5) Quality of included systematic reviews

We assessed the methodological quality of the SRs using a combination of the tool ‘Assessing the Methodological Quality of Systematic Reviews’ (AMSTAR [25]) and the tool ‘Preferred Reporting Items for Systematic Reviews and Meta-Analyses’ (PRISMA‐equity version [26]). PRISMA-equity is a tool to assist reviewers to report on reviews addressing equity issues and provides criteria to assess the reporting quality of SR. These are widely accepted tools to assess the methodological and reporting quality of SR, respectively. Besides assessing the quality of included SRs, we did not reassess the quality of the studies included in those SRs, which were carried out by the SRs’ authors in the first instance.

### 6) Analyses

The units of analyses in this overview were data points or ‘comparisons’, considering together data from the same types of participants, interventions and outcomes. We did not attempt to pool data or carry out meta-analyses due to (i) the large heterogeneity in the types of comparisons; (ii) the frequent lack of data to allow meta-analyses (e.g. number of participants or number of studies) and (iii) the different ways SR reported their findings (e.g. sometimes as pooled estimates and sometimes as single study effects).

To describe the research landscape of systematic reviews addressing equity issues, we present counts and percentages of comparisons by equity attributes.

## Results

**Fig. 1:**
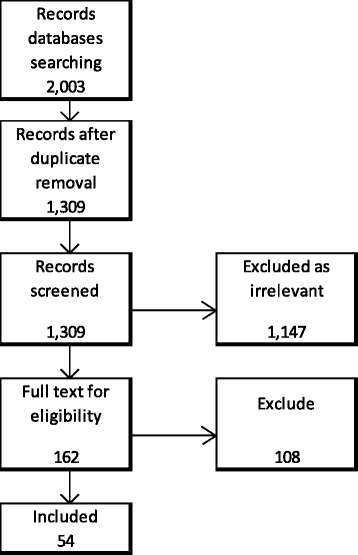
Flow of Systematic Reviews screening and inclusion

The literature databases searches yielded 2,003 hits. After removing 694 duplicates, the remaining 1,309 records were screened for relevance. 162 articles were assessed for inclusion of which 54 were finally included (see Fig. 1). These 54 SRs were those which complied with the inclusion criteria; namely having followed SR methods and reported one vaccination coverage related outcome. The references of included SRs, the characteristics of included SRs and excluded SRs and reasons for exclusion can be found in the Additional file 1: 1 to 3 respectively.

Publication years of included SRs ranged from 1994 to 2014 (median 2008). The oldest study included in the SRs dated from 1950. Studies in SRs were randomised controlled trials (RCT) (29.3%), cluster randomised controlled trials (CRCT) (17.9%) and less frequently controlled before and after (CBA) studies (6.3%) and interrupted time series studies (5.2%). The rest of study designs were observational with and without controls.

SRs and studies within them expanded across all continents. Fifty-six percent of the SRs referred to studies conducted in High Income Countries (HIC), 30% in Middle Income Countries and 14% in Low Income Countries.

The quality of the SRs varied greatly (Table 1). The three most frequent criteria with positive assessments were those reporting on the number of included studies, the sources of studies and the risk of bias method used. The three most frequent criteria with negative assessments were those that lacked (i) a list of excluded studies, (ii) a reporting on the existence of a protocol for the SRs and (iii) information whether or not researchers were contacted.

**Table 1:** Frequencies of the qualtiy criteria of the included Systematic Reviews (from PRISMA and AMSTAR)

PRISMA	Yes		Unclear		No	
SR in title	47	87.0%	1	1.9%	6	11.1%
Rationale	51	94.4%	2	3.7%	1	1.9%
Question	33	61.1%	1	1.9%	20	37.0%
Protocol	14	25.9%	8	14.8%	32	59.3%
Sources	53	98.1%	0	0.0%	1	1.9%
Search	39	72.2%	0	0.0%	15	27.8%
Selection	28	51.9%	3	5.6%	23	42.6%
Data extraction	30	55.6%	1	1.9%	23	42.6%
Researchers contacted	19	35.2%	8	14.8%	27	50.0%
Data variables	27	50.0%	3	5.6%	24	44.4%
Risk of bias method	51	94.4%	0	0.0%	3	5.6%
Type of measures	35	64.8%	2	3.7%	17	31.5%
Synthesis methods	30	55.6%	0	0.0%	24	44.4%
Limitations	33	61.1%	0	0.0%	21	38.9%
Funding	36	66.7%	1	1.9%	17	31.5%
N screened or relevant	49	90.7%	0	0.0%	5	9.3%
N included	53	98.1%	0	0.0%	1	1.9%
Sources grey	19	35.2%	9	16.7%	26	48.1%
List included	43	79.6%	2	3.7%	9	16.7%
List excluded	8	14.8%	2	3.7%	44	81.5%
Conflict of Interest	29	53.7%	2	3.7%	23	42.6%

Table elaborated by the authors based on the quality assessments of Systematic Reviews

Among the 54 SRs, participants were providers (24.1%), caregivers (18.5%), subjects targeted by vaccination (9.3%) or other groups (3.7%). In two other SRs, participants were not described (3.7%). In 22 SRs (40.7%) participants were a combination of the above, including subjects targeted by vaccination.

Twenty‐four of the 54 included SRs reported on more than one intervention. The types of interventions, by decreasing frequency were: ‘processes’ (18.5%), financial, substance or device (7.4%), human resources (3.7%) and organisational interventions (1.9%). The remaining four SRs (7.4%) reported other interventions. Comparators were regular care (46.3%), combination of other interventions (20.4%), financial (3.7%) and process interventions (1.9%). Two SRs reported other comparators (3.7%). Noteworthy, 12 SRs did not report a comparator (22.2%); they included only observational studies.

We extracted 2,568 data points, including effect estimates and individual data points used for those estimates, where available, of which 1,288 reported some type of effect of interventions and considered as ‘comparisons’. The number of data points or comparisons included in this analysis varies because not all the comparisons had complete information on interventions and outcomes.

The most frequently reported outcome was vaccination coverage expressed as rates for a specific vaccine, completion of vaccination schedules or timely vaccination. Knowledge (e.g. about vaccination schedule or other aspects of vaccination) was reported for all vaccine groups except for Human Papilloma Virus (HPV) and Tetanus Toxoid (TT). Morbidity and mortality was reported for the Expanded Programme of Immunisation (EPI) vaccines as well as for other vaccines. Vaccines reported were influenza (44% of data points), vaccines included in the EPI (34%), Pneumococcal Conjugate Vaccine (PCV) (13%), HPV (2%), and TT (1%). Other vaccines (4.6%) included Varicella Vaccine or combinations of the above.

Of the 1,153 comparisons which had effect estimates, effects were reported as favouring the intervention in the vast majority of comparisons: 44.6% and 47.3%, with and without statistically significant estimates, respectively. The control group was favoured in 7.7% of the comparisons and neither positive nor negative effects were reported in 0.4% comparisons.

Of the 54 SRs, 17 (31.5%) explicitly referred to equity groups within their method sections: six of them focused on socio‐economic status (SES), two referred to age groups, six contained a combination of equity attributes and three referred to other categories. However, even reviews which did not explicitly refer to equity in its text might contain data about equity groups (see Table 2). The most common equity group found was ‘place of residence’, which included rural and urban subgroups but also data from different subnational areas, and a miscellaneous group which included, for example, participants vaguely defined as ‘high-risk’, or veterans. SES, occupation, disability and gender were found in slightly more than 20% of SRs. Religion, sexual orientation and education were the least frequently found.

**Table 2:** Number and percentage of Systematic Reviews with data referred to equityattributes

Attributes	Number of reviews with at least 1 factor	
Place of residence	29	53.7%
Race/Ethnicity	10	18.5%
Occupation	15	27.8%
Gender	13	24.1%
Religion	2	3.7%
Education	4	7.4%
SES	16	29.6%
Social capital	5	9.3%
Disability	14	25.9%
Sexual orientation	2	3.7%
Other	29	53.7%
Total	54	100.0%

Table elaborated by the authors based on the data extracted from Systematic Reviews

Looking at more detail on how equity issues are reported in comparisons within SRs, Table 3 represents the relative frequency of each attribute over the whole equity criteria by decade, region and intervention.

**Table 3:** Percentages of data points for each equity attribute, by decade, geographical region and type of intervention

Group	Place of residence	Race, ethnicity	Occupation	Gender	Religion	Education	SES	Social capital	Disability	Sexual orientation	Other	Data points	Data points equity
By decade													
1976 to 1984	20.0%	6.0%	25.0%	0.0%	0.0%	0.0%	14.0%	0.0%	55.0%	0.00%	41.0%	82	51
1985 to 1994	19.0%	2.0%	23.0%	6.0%	0.0%	0.0%	5.0%	0.0%	20.0%	0.00%	45.0%	360	296
1995 to 2004	25.0%	5.0%	12.0%	5.0%	1.0%	1.0%	13.0%	1.0%	26.0%	0.02%	54.0%	633	443
2005 to 2014	18.0%	4.0%	9.0%	17.0%	0.0%	0.0%	13.0%	1.0%	28.0%	0.00%	17.0%	183	174
By region													
Africa	39.0%	2.0%	0.0%	10.0%	0.0%	0.0%	20.0%	2.0%	5.0%	0.00%	10.0%	52	59
Asia	58.0%	0.0%	22.0%	38.0%	4.0%	1.0%	7.0%	4.0%	7.0%	0.00%	19.0%	111	69
Europe	4.0%	2.0%	17.0%	7.0%	0.0%	0.0%	4.0%	0.0%	19.0%	0.00%	42.0%	133	140
LAC	43.0%	0.0%	0.0%	11.0%	0.0%	0.0%	34.0%	0.0%	0.0%	0.00%	2.0%	40	44
North America	22.0%	10.0%	15.0%	4.0%	0.0%	0.0%	14.0%	0.0%	29.0%	0.02%	47.0%	979	689
Oceania	8.0%	0.0%	3.0%	14.0%	0.0%	3.0%	11.0%	0.0%	25.0%	0.00%	44.0%	39	36
By intervention													
Delivery, care	19.0%	5.0%	6.0%	24.0%	0.0%	0.0%	15.0%	0.0%	15.0%	0.00%	33.0%	167	142
Financial	16.0%	1.0%	5.0%	1.0%	0.0%	0.0%	13.0%	0.0%	12.0%	0.00%	22.0%	96	136
Human resources	38.0%	0.0%	0.0%	0.0%	0.0%	0.0%	0.0%	0.0%	0.0%	0.00%	0.0%	3	8
IEC	21.0%	5.0%	16.0%	5.0%	0.0%	0.0%	10.0%	1.0%	21.0%	0.00%	38.0%	767	650
Infrastructures	0.0%	0.0%	0.0%	0.0%	0.0%	0.0%	0.0%	0.0%	0.0%	0.00%	100.0%	4	4
Organisation, regulations	6.0%	3.0%	3.0%	0.0%	0.0%	0.0%	6.0%	0.0%	16.0%	0.00%	3.0%	12	31
Other	21.0%	10.0%	23.0%	3.0%	0.0%	2.0%	10.0%	0.0%	27.0%	0.00%	34.0%	404	311
Substance/device	100.0%	0.0%	0.0%	100.0%	0.0%	0.0%	0.0%	0.0%	0.0%	0.00%	100.0%	9	3

Sums of rows may add up to more than 100% because more than one equity group may be reported in the same comparison

Table elaborated by the authors based on the data extracted from Systematic Reviews

There does not seem to be a clear overall tendency of an equity attribute to stand out over the others by decade, except for the gender attribute which seemed to be more prominent in the last decade (in 17% comparisons) than in previous decades (0.0 to 6.0%). Contrarily, two attributes seemed less frequently reported in the last decade: occupation and the ‘other’ attribute. It has to be noted that the number of data points in the last decade was lower than before, reflecting the time lapse required to incorporate recent research into SRs.

The most frequently reported attribute was ‘place of residence’ in SRs with studies from Africa, Latin America and Caribbean and in Asia. On the other hand, ‘disability’ was the most frequent (‘other’ apart) in North America, Europe and Oceania. The few comparisons involving sexual orientation were from North America and the few on religion from Asia. The relative frequency of ‘gender’ over all groups was higher in Asia than in other regions.

Looking at the type of intervention, the most common comparisons were related to the delivery of care, financial interventions, IEC and a miscellaneous group. Place of residence was the first or second most frequently encountered attribute in these comparisons in all types of interventions. Predominant groups in the delivery of care interventions were gender, ‘place of residence’, SES and disabilities. IEC interventions were implemented according to the ‘place of residence’, in people with disabilities, in occupation subgroups and in others. As could be expected, the most frequent group for financial interventions was the SES.

The great majority of SRs and studies within them did not estimate differentials or gradients across equity attributes, except for a few studies. Usman 2009 (in SR Kaufman 2013) was a RCT on the effects of face-to-face interventions to improve diphtheria-tetanus-pertussis coverage in Pakistan. The study compared urban and rural areas with Odds Ratios (OR) of 1.18 (95CI: 1.05 to 1.33) and OR 1.54 (95CI: 1.33 to 1.79) favouring the interventions, respectively. La Montaigne 2011 (in SR Paul 2014) was an observational study in India comparing the third dose of HPV vaccine coverage in urban, rural and tribal areas, with before and after changes ranging from 68.1 to 77.2%, from 83.3 to 87.8% and from 71.1 to 68.1%, respectively. Cuttes 1988 (in SR Ryman 2008) was another observational study in Mozambique assessing the effects of visiting homes to mobilise the community on fully vaccinated children in four areas of residence, with changes in coverage ranging from −4 to 33%. Cutts 1990 (in SR Shea 2009) was a study using different methodological approaches assessing outreach, communication, training and volunteers activities to improve measles vaccination in four locations, showing changes in coverage ranging from 1 to 31%.

## Discussion

We have carried out an overview of SRs containing information related to vaccination, looking for equity attributes as explicitly described by authors or as found using the PROGRESS-plus groups. We examined the area of vaccination under a very wide perspective not limiting SRs according to study designs, participants, interventions or outcome, which gives a comprehensive view of a key public health area. We are not aware of any other global overview describing how research has reported evidence on interventions to improve vaccination outcomes from an equity perspective, although there are examples of SRs on interventions to reduce inequities in specific groups, such as immigrants [27], or through health systems interventions [28], or studies describing the distribution of equity attributes among populations [5, 29]. Research evidence predominates in certain HIC, mainly in relation to influenza vaccination, addressing a limited scope of equity attributes and with scarcity of studies which show changes in equity gaps or gradients between population subgroups.

This overview suggests that equity attributes were widely but inconsistently represented in the scientific literature. There was a predominance of research carried out in North America with many studies focusing on influenza vaccines. These studies did not seem particularly more focused on equity issues as compared with studies from other regions or with other vaccines. However, research on vaccines administered at different ages (e.g. routine childhood vaccination as compared to influenza vaccination) may uncover age-specific challenges in accessing vaccination; similarly with different countries and settings. These differences did not particularly emerge in this overview of SRs, which could be precisely due to the fact that research is not really focusing on equity attributes but rather on subjects who happened to belong to different subgroups of population.

The identification of equity attributes may be due to the fact that equity has been actively promoted by the international community [30]. We would therefore expect an increased awareness on equity among researchers in recent years, which could have been captured by the fact of not having put any time limit in the search for SRs. However, this has not necessarily led to uniform improvements in equity in the last decade, especially in LMIC [31, 32]. Additionally, the PROGRESS-plus groups cover such a very wide range of situations that it should not be difficult to find studies and SRs on equity attributes. This is not striking as such because, most of the population in the globe are living in LMIC or are poor [33, 34] and it is not difficult to find a study which involves these sectors of the population. However in our overview, besides the attributes ‘place of residence’ and ‘other’, the presence of equity attributes was much more modest and severely scarce in some. Research from LMIC where much of the burden of disease is [35] and where vaccination coverage rates are lower [36], is less abundant and not particularly focused on equity. It is striking for example, that despite consistent evidence that the level of education of caregivers is negatively related to vaccination status in LMIC [37], education is one of the least addressed equity factors we could find. One explanation for this could be the ethical dilemmas of carrying out comparative studies in which one control or comparison group would not benefit from a basic educational intervention, such as literacy education.

Inequity is not about being in a given level of SES or in a gender group, but a matter of ‘differences’ and has an intrinsic ethical dimension; it is about justice [38, 39]. Actually inequities point to the fact that disadvantages in some human groups are directly related to privileges in other groups. Targeted interventions may have an impact on improving the health of vulnerable populations [12]; yet, this does not necessarily imply reducing differences and improving equity. In most of the evidence we retrieved, interventions had vulnerable groups as participants, but they did not seem to be specifically designed to address the gaps in health and health care between disadvantaged and privileged groups, as reported by authors. Studying the poor or the vulnerable, being essential and desirable, does not necessarily provide evidence on the key issues of how inequities can be reduced [40], or how health and health care resources can be better redistributed. It is striking that examples of analyses of group differences, which could very well inform approaches to better redistribute health care resources, i.e. estimates of effects between privileged and vulnerable populations, were hardly found and those that were, were of a reduced scope. While some disadvantaged groups are the largest majority, other vulnerable populations are actually minorities, e.g. transsexual people [41] or prisoners [42], and as expected they are much less present in the research we examined. The fact that some of these disadvantaged groups are actually minorities may be a contributing factor for not having been extensively studied..

We acknowledge that research on equity may pose methodological challenges [43], which could explain the relative lack of comparators in a large proportion of the underlying (observational) studies, confirmed in the findings of a recent review [44]; for example, larger sample sizes may be needed to allow comparisons between subgroups or study settings and implementation may need more resources and time. Mortality and life expectancy differentials, as proposed to monitor SDG [45], are examples of methodological challenges which may contribute to ‘discourage’ the equity focus in indicators related to universal health coverage [46].

Furthermore, the heterogeneity of approaches limits the capacity to compare strategies to reduce inequity in different health care fields and contexts. Therefore, equity definitions such PROGRESS-plus need to be urgently complemented with the development of robust and realistic methods to measure inequities in health care [47] both at macro and micro levels. Besides, agreement across research areas on critical equity outcomes would benefit further efforts to conduct research synthesis on inequities [48].

Another issue to be considered is that critical equity attributes may be different in nature and importance in different types of health problems. In the area of vaccination, the age groups and settings to deliver influenza vaccine greatly differ from routine childhood vaccinations. This is also more the case in other health areas. For example, children and the productive age group have been associated with fatalities in road traffic accidents, especially in LMIC.

Our overview had several limitations. First, inclusion criteria and data extraction was single-applied, which may have increased the chances of errors, which we cannot exclude despite the controlling mechanisms we put in place while carrying out the work. However, we believe that this could hardly have any impact in the main conclusions of this overview. Neither could we rule out duplicate reporting if primary studies were included in more than one of the included SRs, since some of the SRs did not contain a full list of references. Secondly, we actually found poor reporting in SRs and in the studies contained in them and this was our subjective impression when reading the articles. Poor reporting jeopardises the capacity to produce sound evidence on equity, which requires some additional details in the description of interventions, outcomes and implementation approaches.

## Conclusion

Equity (and inequities) is a top priority issue in the human health development agenda [49] and an entry point to install clear ethical directions in the redistribution of health and health care. Often, it is not the most marginalised that receive assistance, as indicated in a recent overview on official development assistance [50]. While the amount of descriptive research on the status of the most vulnerable population is overwhelming and there is also abundant research on interventions to improve vaccination outcomes, our overview identified two gaps: (a) research on specific minority groups (e.g. religious or groups defined by sexual orientation) and (b) research on what works, not only to improve health outcomes in general, but to close the inequity gaps between the privileged and the vulnerable populations: how to effectively redistribute health among the different layers or society. This is essential to inform national and international choices which affect the access of the most vulnerable (being they majorities or minorities) to the health care that others already enjoy. Research and policy, if wishing to remain relevant, should mirror the unbearable suffering of disadvantaged human beings by prioritising the production of robust evidence on what affects them, no matter how many they are or where they live.

## Additional file

Additional file 1: Ovid MEDLINE(R) In-Process & Other Non-Indexed Citations, Ovid MEDLINE(R) Daily, Ovid MEDLINE(R) and Ovid OLDMEDLINE(R) 1946 to Present. (DOCX 13 kb)

## References

[1] Braveman P. What Are Health Disparities and Health Equity? We Need to Be Clear. Public Health Rep. 2014;129(Suppl 2):5–8. 10.1177/00333549141291S203 PMC3863701 24385658

[2] Whitehead M (1992). The concepts and principles of equity and health. Int J Health Serv.

[3] United Nations. Transforming our world: the 2030 Agenda for Sustainable Development. New York: United Nations; 2015.

[4] WHO. Global and regional immunisation profille - Global. Version 2016-Nov-18.

[5] WHO. Global and regional immunisation profile – African Region. Version 2016-Nov-18.

[6] Barros AJ (2012). Equity in maternal, newborn, and child health interventions in Countdown to 2015: a retrospective review of survey data from 54 countries. Lancet.

[7] Bosch-Capblanch X, Banerjee K, Burton A (2012). Unvaccinated children in years of increasing coverage: how many and who are they? Evidence from 96 low- and middle-income countries. Trop Med Int Health.

[8] Delamonica E, Minujin A, Gulaid J (2005). Monitoring equity in immunization coverage. Bull World Health Organ.

[9] Save the Children. Further, Faster and Fairer. The Save the Children Fund 2016.

[10] World Health Organization. Global vaccine action plan 2011–2020, World Health Organization, Editor. Geneva; 2013.

[11] Brearley L (2013). Applying an equity lens in the decade of vaccines. Vaccine.

[12] Bar-Zeev N, Tate JE, Pecenka C, Chikafa J, Mvula H, Wachepa R, Mwansambo C, Mhango T, Chirwa G, Crampin AC, Parashar UD, Costello A, Heyderman RS, French N, Atherly D, Cunliffe NA, VACSURV Consortium (2016). Cost-effectiveness of monovalent rotavirus vaccination of infants in Malawi: a postintroduction analysis using individual patient-level costing data. Clin Infect Dis.

[13] La Rosa E, Dubois G, Tonnellier F. [Social responsibility in health and the global health situation: towards new health and social indicators]. Santé Publique. 2007;19(3):217–27. 10.3917/spub.073.0217 17708486

[14] Broutet N, Lehnertz N, Mehl G, Camacho AV, Bloem P, Chandra-Mouli V, Ferguson J, Dick B (2013). Effective health interventions for adolescents that could be integrated with human papillomavirus vaccination programs. J Adolesc Health.

[15] Rahkumar S, Bosch-Capblanch X. Key elements of national immunisation policies. Swiss TPH. January 2014. Unpublished report submitted to 3 Impact Evaluation.

[16] Valentine NB, Bonsel GJ (2016). Exploring models for the roles of health systems’ responsiveness and social determinants in explaining universal health coverage and health outcomes. Glob Health Action.

[17] Oyo-Ita A, Nwachukwu CE, Oringanje C, Meremikwu MM (2011). Interventions for improving coverage of child immunization in low- and middle-income countries. Cochrane Database Syst Rev.

[18] Jacobson Vann JC & Szilagyi P. (2005). Patient reminder and recall systems to improve immunization rates. Cochrane Database Syst Rev. (3). doi:10.1002/14651858.CD003941.pub2. 10.1002/14651858.CD003941.pub2 PMC6485483 16034918

[19] Kaufman J, Synnot A, Ryan R, Hill S, Horey D, Willis N, Robinson P. (2013). Face to face interventions for informing or educating parents about early childhood vaccination. Cochrane Database of Systematic Reviews, (5). doi:10.1002/14651858.CD010038.pub2. 10.1002/14651858.CD010038.pub2 23728698

[20] Målqvist M, Yuan B, Trygg N, Selling K, Thomsen S. Targeted interventions for improved equity in maternal and child health in low- and middle-income settings: a systematic review and meta-analysis. PLoS One. 2013;8(6):e66453. 10.1371/journal.pone.0066453 PMC3688766 23840474

[21] WHO: Immunization, Vaccines and Biologicals. Immunization Coverage. 2017.http://www.who.int/immunization/monitoring_surveillance/routine/coverage/en/. Accessed 5 Apr 2017.

[22] PDQ-Evidence: Evidence for Informed Health Policymaking. 2014. http://www.pdq-evidence.org/. Accessed Nov 2014.

[23] Tugwell, P., et al., Assessing equity in systematic reviews: realising the recommendations of the Commission on Social Determinants of Health. BMJ. 2010;341:c4739. doi:10.1136/bmj.c4739. 10.1136/bmj.c4739 20837575

[24] O’Neill J (2014). Applying an equity lens to interventions: using PROGRESS ensures consideration of socially stratifying factors to illuminate inequities in health. J Clin Epidemiol.

[25] AMSTAR: AMSTAR Checklist. (2015)http://amstar.ca/Amstar_Checklist.php. Accessed August 2016.

[26] Welch V, Petticrew M, Petkovic J, Moher D, Waters E, White H, Tugwell P, PRISMA-Equity Bellagio Group. Extending the PRISMA Statement for equity-focused systematic reviews (PRISMA-E 2012): Explanation and elaboration. J Clin Epidemiol. 2015. [Epub ahead of print] 10.1016/j.jclinepi.2015.09.001 26348799

[27] Batista R, Pottie K, Bouchard L, Ng E, Tanuseputro P, Tugwell P. Primary Health Care Models Addressing Health Equity for Immigrants: A Systematic Scoping Review. J Immigr Minor Health. 2016. 10.1007/s10903-016-0531-y 27858278

[28] Sumah AM, Baatiema L, Abimbola S (2016). The impacts of decentralisation on health-related equity: A systematic review of the evidence. Health Policy.

[29] Hosseinpoor AR, Bergen N, Schlotheuber A, Gacic-Dobo M, Hansen PM, Senouci K, Boerma T, Barros AJ (2016). State of inequality in diphtheria-tetanus-pertussis immunisation coverage in low-income and middle-income countries: a multicountry study of household health surveys. Lancet Glob Health.

[30] Marmot M, Friel S, Bell R, Houweling TA, Taylor S (2008). Commission on social determinants of health. Closing the gap in a generation: health equity through action on the social determinants of health. Lancet.

[31] Boerma JT, Bryce J, Kinfu Y, Axelson H, Victora CG, Countdown 2008 Equity Analysis Group (2008). Mind the gap: equity and trends in coverage of maternal, newborn, and child health services in 54 Countdown countries. Lancet.

[32] Alkenbrack S, Chaitkin M, Zeng W, Couture T, Sharma S (2015). Did equity of reproductive and maternal health service coverage increase during the MDG Era? an analysis of trends and determinants across 74 Low- and middle-income countries. PLoS ONE.

[33] World Bank: Population ranking. http://data.worldbank.org/data-catalog/Population-ranking-table (2016). Accessed August 2016.

[34] World Bank: Poverty overview. http://www.worldbank.org/en/topic/poverty/overview (2016). Accessed August 2016.

[35] Global Burden of Disease Study 2013 Collaborators (2015). Global, regional, and national incidence, prevalence, and years lived with disability for 301 acute and chronic diseases and injuries in 188 countries, 1990–2013: a systematic analysis for the Global Burden of Disease Study 2013. Lancet.

[36] Subaiya S et al. Global Routine Vaccination Coverage, 2014. Morb Mortal Wkly Rep. 2015:64(44);1252–55. 10.15585/mmwr.mm6444a5 26562454

[37] Merten S, Martin Hilber A, Biaggi C, Secula F, Bosch-Capblanch X, Namgyal P, Hombach J (2015). Gender determinants of vaccination status in children: evidence from a meta-ethnographic systematic review. PLoS One.

[38] WHO: Health Inequality and Inequity. Glossary. http://www.who.int/hia/about/glos/en/index1.html (2016). Accessed 11 Aug 2016.

[39] Melamed C, Samman E (2015). Equity, inequality and human development in a post.

[40] Bull World Health Organ 2009;87:84 | doi:10.2471/BLT.08.062695. Accessed Aug 2016.

[41] Grossman AH, D’augelli AR (2006). Transgender youth: Invisible and vulnerable. J Homosex.

[42] WHO: Prisons and Health. World Health Organization, Regional Office for Europe. http://www.euro.who.int/__data/assets/pdf_file/0005/249188/Prisons-and-Health.pdf (2014). Accessed 5 Jun 2016.

[43] Bauer GR (2014). Incorporating intersectionality theory into population health research methodology: Challenges and the potential to advance health equity. Soc Sci Med.

[44] Crocker-Buque T, Edelstein M, Mounier-Jack S. Interventions to reduce inequalities in vaccine uptake in children and adolescents aged <19 years: a systematic review. J Epidemiol Community Health. 2016. doi: 10.1136/jech-2016-207572. [Epub ahead of print] Review. 10.1136/jech-2016-207572 PMC5256276 27535769

[45] WHO (2015). Towards a monitoring framework with targets and indicators for the health goals of the post-2015 Sustainable Development Goals.

[46] Chapman AR. The Problems with the Proposed Indicators for Monitoring Universal Health Coverage in the Sustainable Development Goals. Health and Human Rights Journal. 2016.

[47] Tugwell P, Robinson V, Morris W (2007). Mapping global health inequalities: challenges and opportunities. eScholarship Repository.

[48] Nantulya VM, Reich MR (2002). The neglected epidemic: road traffic injuries in developing countries. BMJ.

[49] Tangcharoensathien V, Kanchanachitra C, Thomas R, Headen Pfitzerd J, Whitneye P (2016). Addressing the health of vulnerable populations: a call for papers. Bull World Health Organ.

[50] The Economist: Misplaced Charity - Aid is best spent in poor, well-governed countries. That isn’t where it goes. The Economist, 11 June 2016. http://www.economist.com/news/international/21700323-development-aid-best-spent-poor-well-governed-countries-isnt-where-it. Accessed 13 Jun 2016.

