# Supplementary material for: Research on subgroups is not research on equity attributes: Evidence from an overview of systematic reviews on vaccination
Source: Int J Equity Health. 2017 Jun 7;16:95. doi: 10.1186/s12939-017-0587-x (PMC5463415; doi:10.1186/s12939-017-0587-x)
Supplement: Additional file 1: — Ovid MEDLINE(R) In-Process & Other Non-Indexed Citations, Ovid MEDLINE(R) Daily, Ovid MEDLINE(R) and Ovid OLDMEDLINE(R) 1946 to Present. (DOCX 13 kb) [file 12939_2017_587_MOESM1_ESM.docx]

**Ovid MEDLINE(R) In-Process & Other Non-Indexed Citations, Ovid MEDLINE(R) Daily, Ovid MEDLINE(R) and Ovid OLDMEDLINE(R) 1946 to Present**

1. exp Vaccines/ad, ec, lj, st, sd, tu [Administration & Dosage, Economics, Legislation & Jurisprudence, Standards, Supply & Distribution, Therapeutic Use]

2. immunotherapy/ or immunization/ or exp immunization, passive/ or immunization schedule/ or immunization, secondary/ or exp immunotherapy, active/

3. exp Immunization Programs/cl, ec, es, lj, ma, mt, og, st, sn, sd, td, ut [Classification, Economics, Ethics, Legislation & Jurisprudence, Manpower, Methods, Organization & Administration, Standards, Statistics & Numerical Data, Supply & Distribution, Trends, Utilization]

4. (vaccin* or revaccinat* or immuniz* or immunis* or immunotherap* or inoculat* or innoculat*).tw.

5. 1 or 2 or 3 or 4

6. (systematic review or meta analy* or metaanaly* or metanaly*).ti,hw,pt.

7. 5 and 6

8. exp Animals/

9. Humans/

10. 8 not (8 and 9)

11. 7 not 10

12. limit 11 to yr="2013 -Current"
